# Direct field method for root biomass quantification in agroecosystems

**DOI:** 10.1016/j.mex.2016.08.002

**Published:** 2016-08-04

**Authors:** Ileana Frasier, Elke Noellemeyer, Romina Fernández, Alberto Quiroga

**Affiliations:** a Instituto Nacional de Tecnología Agropecuaria, EEA Anguil, La Pampa, Argentina; b Consejo Nacional de Investigaciones Científicas y Técnicas, Argentina; c Facultad de Agronomía Universidad Nacional de La Pampa, Santa Rosa, La Pampa, Argentina

**Keywords:** Direct field method for root biomass quantification in agroecosystems, row crops, soil auger, root washing, root mass per area

## Abstract

The present article describes a field auger sampling method for row-crop root measurements. In agroecosystems where crops are planted in a specific design (row crops), sampling procedures for root biomass quantification need to consider the spatial variability of the root system. This article explains in detail how to sample and calculate root biomass considering the sampling position in the field and the differential weight of the root biomass in the inter-row compared to the crop row when expressing data per area unit. This method is highly reproducible in the field and requires no expensive equipment and/or special skills. It proposes to use a narrow auger thus reducing field labor with less destructive sampling, and decreases laboratory time because samples are smaller. The small sample size also facilitates the washing and root separation with tweezers. This method is suitable for either winter- or summer crop roots.

• Description of a direct field method for row-crop root measurements.
• Description of data calculation for total root-biomass estimation per unit area.
• The proposed method is simple, less labor- and less time consuming.

## Graphical abstract

**Figure:**
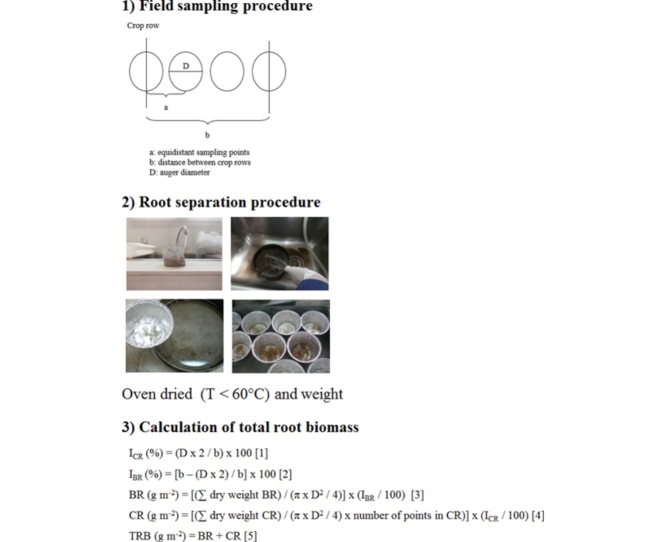

## Method details

### Field sampling procedure

**Fig. 1:**
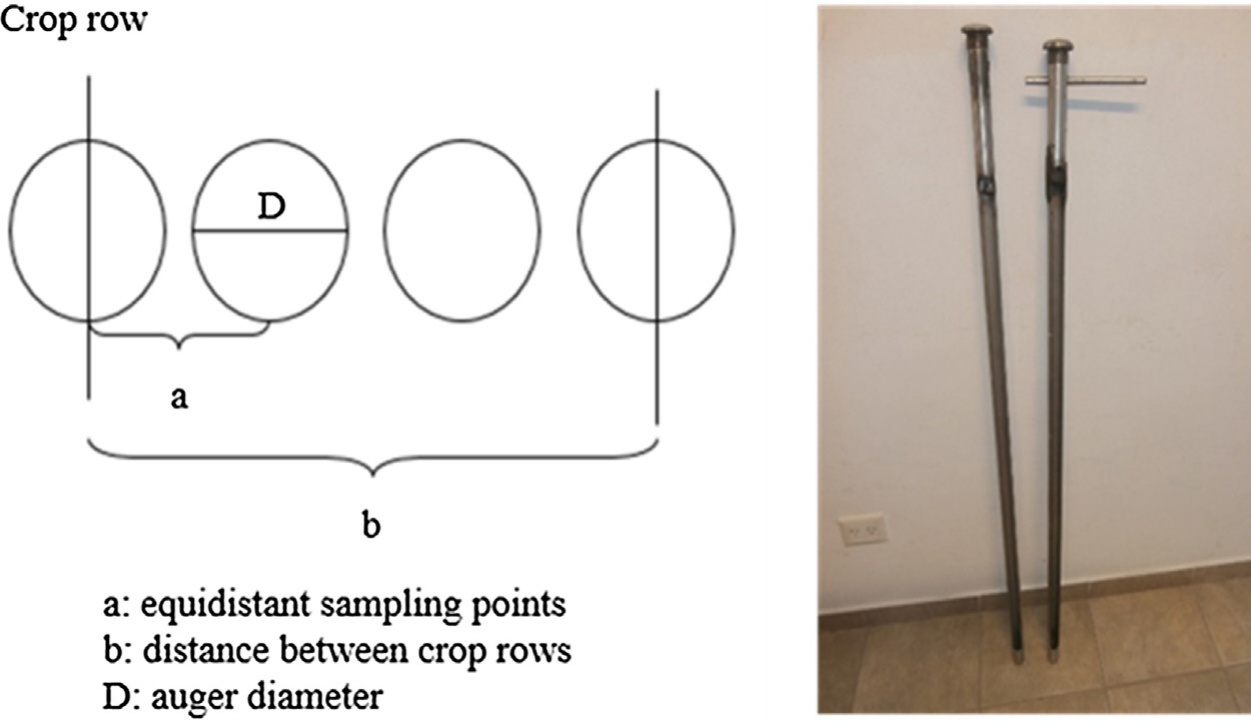
Diagram representing the root sampling in row crops and the auger type used.

In the field, take four samples at equidistant points in between two crop rows with a narrow tubular soil auger (0.032 m diameter). The first and last sampling points have to coincide with two neighboring crop rows (Fig. 1). For determination of the equidistant points, it is convenient to use a ruler or metric tape. In very sandy soils, first take the samples on the crop-rows and then those in-between rows. In order to avoid soil crumbling and drift when introducing the auger the soil can be moistened, or sampling should be carried out when soil has good moisture conditions.

The objectives of each study and the length of the available auger will define the overall sampling depth. However, this method is especially recommendable for rooting depth and root stratification studies. It is highly recommendable to take at least four full replicates for each experimental unit (e.g. plot), thus reducing the inherent spatial variability and accounting for increased variability with depth (Table 1). When there are few roots present in the between-row samples, these can be pooled into one sample per depth interval for further study, or they will have to be processed individually if there are abundant roots. For field plots without vegetation, e.g. fallow, random sampling or the same procedure as described above can be used. Immediately after taking the soil samples place these into plastic bags and keep in a freezer at − 20 °C until washing.

**Fig. 2:**
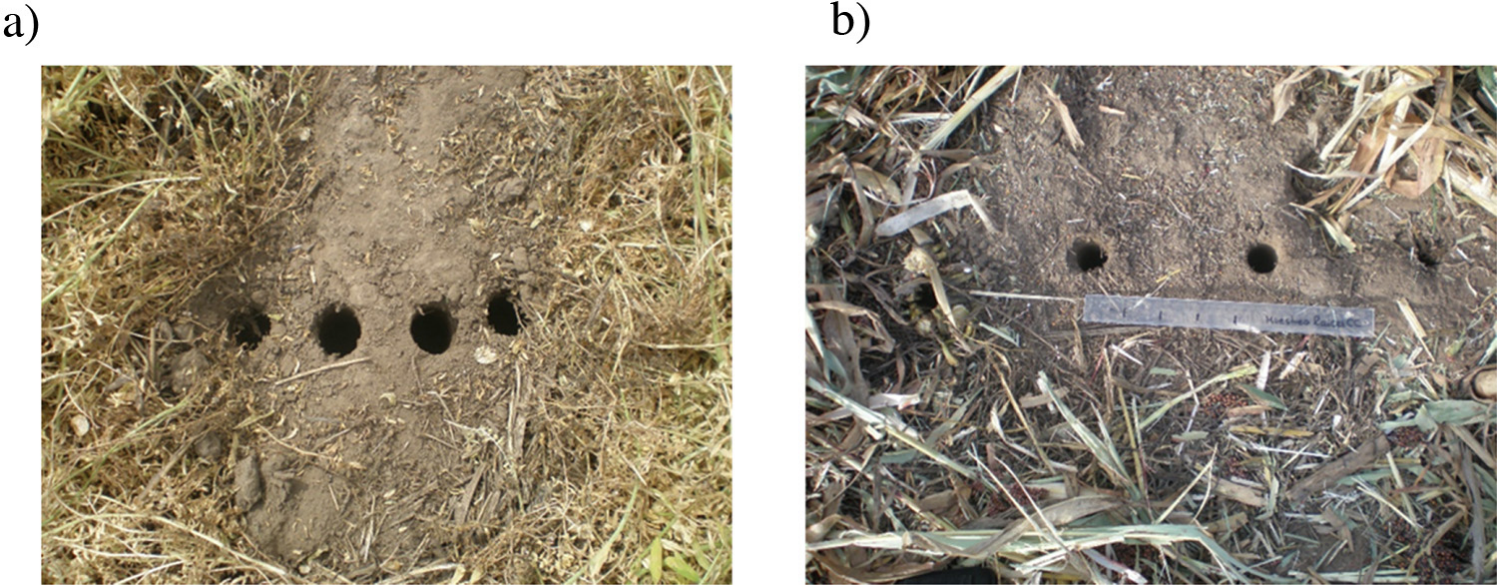
Examples of roots field sampling for vetch (a) and sorghum (b) with a distance between rows of 0.17 and 0.52 m respectively.

The proposed method is valid and useful for studying both winter and summer crops (Fig. 2). Time-series of root determinations within the same plot are useful to analyze the effect of crop rotations on root dynamics [2].

### Root separation procedure

**Fig. 3:**
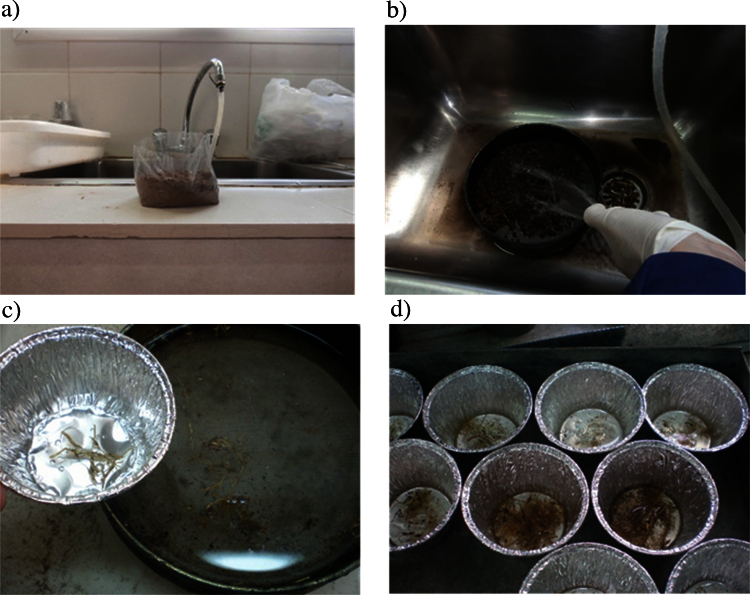
Steps for root separation from soil: a: sample, b: washing step, c: recollection of roots with tweezers, d: roots from crop-row and between-row points prior to oven drying.

In order to separate roots from soil wash the samples through a submerged 250 μm sieve with running tap water [3] and then collect the roots retained by- and floating on the sieve with tweezers (Fig. 3). The recuperation of roots depends on sieve mesh size [4], therefore it is necessary to unify criteria in order to obtain comparable results. Processing small-sized samples facilitates root recollection by flotation since this allows for using smaller mesh sizes independent of soil texture.

Root samples are then oven-dried to constant weight at temperatures below 60 °C and weighed using a precision scale. The dry root material should be stored in well-closed bags or plastic vials in a dry place. These samples can be used to determine root length by image analysis [5], or can be milled for chemical analysis of the root biomass.

## Calculation of total root biomass

Considering the differential weight of the root biomass in the inter-row per area unit compared to the root biomass in the row is crucial for obtaining representative results. This is done by calculating the influence-percentage (I%) using the data for the distance between crop-rows (b) and the diameter of the auger (D). The following equations use the parameters of the diagram shown in Fig. 1.

(1) *I_CR_ (%)* *=* *(D × 2/b)* × *100*

(2) *I_BR_ (%)* *=* *[*(*b − (D* × *2)/b]* × *100*

Here we proceed to calculate between-row root biomass. The principle is to consider the dry weight of roots in the section occupied by the between-row area (π x D/4) corrected by the percentage that this section occupies in one hectare.

(3) *BR (*g m^−2^*)* *=* *[(∑ dry weight BR)/(π* × *D^2^/4)]* × *(I_BR_/100)*

In a similar manner, calculate the crop-row root biomass, for which the average weight of roots for both crop-row sampling points is used in order to represent crop-row root biomass.

(4) *CR (*g m^−2^*)* *=* *[(∑ dry weight CR)/(π* × *D^2^/4* × *number of points in CR)]* × *(I_CR_/100)*[4]

The sum of crop-row and between-row root biomass represents the total root biomass (TRB).

(5) *TRB (*g m^−2^*)* *=* *BR* *+* *CR*

Total root biomass data must be transformed to logarithm to ensure data normal distribution for ANOVA analysis.

## Additional information

Crop root biomass data are scarce and are often calculated from aerial biomass by applying a root-shoot coefficient [6]. However, soil models need to include correct data of root- as well as mineral- and microbial carbon to make accurate global carbon projections. Root to shoot ratios are not a sufficiently precise measurement for carbon stock estimations due to the considerable variability encountered in the data [7]. Although many sophisticated techniques are now available [8], [9], [10], [11], [12], [13], [14], direct field methods are more common because they need no special equipment or skills, and are easy, fast, and inexpensive to use [15]. The auger methods are most suitable for taking volumetric soil-root samples [16] and are specially recommended for accounting for the spatial variability of fine-root distribution [17]. Rooting depth can be easily studied with this method when other expensive equipment, for example a rhizotron, is not available [18], [19].

In agroecosystems where crops are planted in a specific design (row crops), sampling procedures for root biomass quantification need to consider the spatial variability of the root system. In fact, there is no consensus in the literature about the correct field sampling procedure and the criteria for root biomass calculation. In some studies samples were taken only in the inter-row [20], [21], while others consider different positions (e.g. within and between rows) but use an average value as root biomass estimator [6], [22], [23]. Moreover, there are other cases were field sampling method is not described at all [24], [25]. If samples were only taken in the inter-row this could lead to underestimating total root biomass while calculating the influence-percentage (I%) of roots in crop-row (CR) and between-row (BR) could be a more precise way to estimate total biomass (Table 2). In a similar way, averaging of data from crop-row and between-row as suggested by some authors [6], [22], [23] would lead to an overestimation of root biomass (Table 2).

## Concluding remarks

The proposed new method to measure root biomass in row crops has several advantages over many traditional ways of root quantification. It is a simple and rapid field method that requires neither special equipment nor trained personnel, it needs no specific laboratory materials, and it takes into account the spatial variability of roots in row crops. Thus, we recommend this method for accurate estimation of root biomass in field studies or carbon stock surveys in agroecosystems.

## Figures and Tables

**Table 1:** Root biomass and its variation coefficient (%) for different winter crops (R-rye, V-vetch, VR- vetch-rye association) in two contrasting soils (Ustipsamment and Paleustoll [1]) at different depths. Variation coefficient was calculated as a ratio between standard deviation and arithmetic mean. Means correspond to four replicates per plot. SD: standard deviation.

		Ustipsamment			Paleustoll		
Depth	Species	Mean	SD	CV (%)	Mean	SD	CV (%)
0–0.20 m	R	428	51	12	235	38	16
	V	194	17	9	231	37	16
	VR	364	62	17	325	107	33

0.20–0.40 m	R	27	2	8	40	8	21
	V	36	4	11	30	7	23
	VR	13	2	15	55	19	35

0.40–0.60 m	R	14	0.4	3	38	3	7
	V	27	11	41	28	4	13
	VR	14	5	33	44	17	38

0.60–0.80 m	R	11	5	45	54	22	41
	V	30	14	48	56	24	42
	VR	13	1	7	42	11	27

0.80–1 m	R	9	4	45	54	22	41
	V	24	12	49	56	25	45
	VR	10	1	8	42	11	27

**Table 2:** Comparison of different methods for root measurement (g m^−2^). Average method means the average of crop-row and between-row data. Intercrop represents only data from the between-row samples. The new methods represents the data obtained by the method proposed. Means correspond to four replicates of different winter crops (R-rye, V-vetch, VR- vetch-rye association) in two contrasting soils (Ustipsamment and Paleustoll [1]) at different depths.

		Ustipsamment			Paleustoll		
Depth	Species	Average method^a^	Intercrop sampling^b^	New method^c^	Average method	Intercrop sampling	New method
0–0.20 m	R	694	169	428	367	158	235
	V	297	158	194	353	203	231
	VR	576	213	364	511	202	325

0.20–0.40 m	R	40	26	27	61	33	40
	V	53	43	36	45	25	30
	VR	20	12	13	81	60	55

0.40–0.60 m	R	21	13	14	58	32	38
	V	40	30	27	42	26	28
	VR	21	14	14	65	46	44

0.60–0.80 m	R	16	10	11	81	52	54
	V	47	25	30	82	66	56
	VR	19	13	13	63	39	42

0.80–1 m	R	13	8	9	81	52	54
	V	37	20	24	82	66	56
	VR	15	10	10	63	39	42

a Average of all sampling points in the crop row and between rows.

b BR (g m^−2^) = [(∑ dry weight BR)/(π × D^2^/4)].

c New method proposed according to Eqs. (1)–(5).
